# Data-driven pit stop decision support for Formula 1 using deep learning models

**DOI:** 10.3389/frai.2025.1673148

**Published:** 2025-11-05

**Authors:** Abhijai Sasikumar, A. Anny Leema, P. Balakrishnan

**Affiliations:** School of Computer Science and Engineering, Vellore Institute of Technology, Vellore, Tamil Nadu, India

**Keywords:** Bidirectional Long Short-Term Memory (Bi-LSTM), deep learning, Formula 1, pit stop strategy, race data visualization, Synthetic Minority Over-sampling Technique (SMOTE), telemetry data, time series classification

## Abstract

In Formula 1, which is among the most competitive motorsports in the world, the timing of a pit stop can make the difference between winning and losing a race. Conventional methods based on human judgment can be erratic, especially in rapidly changing race conditions. This work proposes a datadriven framework based on deep learning models to predict optimal pit stop timings using raw telemetry data extracted from FastF1 API. To improve the robustness of the models, advanced preprocessing techniques such as normalization, imputation, and class balancing with Synthetic Minority Over-sampling Technique (SMOTE) were implemented. Five different deep learning architectures, including Bi-LSTM, TCN-GRU, GRU, InceptionTime, and CNN-BiLSTM, were trained and evaluated employing precision, recall, and F1-score as metrics. Of these, the Bi-LSTM model achieved the overall best performance which can be explained by its capability to model long-range dependencies in both forward and backward temporal directions. The Bi-LSTM achieved a precision of 0.77, recall of 0.86, and an F1-score of 0.81 on the test set, demonstrating strong predictive accuracy under real-race conditions. Additionally, a historical race visualization interface was developed to visualize the model's predictions.

## 1 Introduction

Formula 1 is the pinnacle of motorsports. However, contrary to general assumption, it involves much more than just being the fastest on the track. Formulating a data-driven race strategy is quintessential. Each year, all the ten teams pour millions of dollars in the quest to secure the Drivers' and Constructors' championships. Optimizing the timing of a pit stop, which is a precisely timed intervention that predominantly includes the change of the tire compound to a different one according to the race conditions, can be a game changer.

The strategists across the ten teams work round the year in order to optimize the race strategies, specifically to reduce lap times which sometimes boil down to even one-thousandth of a second. Varying track conditions and unpredictable weather conditions make the task even more difficult. Hence, relying on past patterns alone would not suffice.

Leveraging artificial intelligence can reveal underlying patterns that often go unnoticed by human strategists. This work presents a data-driven framework for predicting optimal pit-stop windows in Formula 1. Historical race data from 2020–2024 were extracted using the FastF1 API, and modeled with deep-learning architectures—Bi-LSTM, TCN-GRU, GRU, InceptionTime and CNN-BiLSTM. Comprehensive pre-processing (normalization, imputation, and class balancing) is applied to maximize model performance. The models are then compared across multiple metrics to highlight their respective strengths and weaknesses, and visual comparisons illustrate the competitive advantage offered by the AI-based insights.

A Formula 1 grid normally comprises 20 cars—two entries from each of the ten teams. Points are awarded to the top-ten finishers, from 25 points for the winner, 18 for second, down to 1 point for tenth. A poorly timed pit stop can drop a driver several places when cars are separated by razor-thin margins; conversely, a well-timed pit stop can yield critical track-position gains. Such small improvements have two effects: in the short term, they can secure a podium finish; in the long term, they accumulate extra points that lift a team up the Constructors' Championship leaderboard. At season's end, prize money is distributed according to those standings—the higher the position, the larger the payout. Notably, moving from ninth to eighth in the table is worth roughly USD 10 million. By providing accurate, real-time pit-stop predictions, the proposed framework converts raw telemetry data into actionable strategy decisions and enhances competitive performance across teams.

## 2 Related works

Heilmeier et al. (2020) proposed a Virtual Strategy Engineer (VSE) that uses a combination of a Feed Forward Neural Network (FFNN) and a Long Short Term Memory (LSTM) in order to make automated race strategy decisions. The VSE focused on optimizing pit stop timing and selecting the correct tire compound. The strength of the VSE lay in adapting to multiple adverse race conditions such as yellow and red flags. When simulated with the data of the 2019 Chinese Grand Prix, the VSE achieved an average finishing position of 9.51 across 1,000 simulations. The limitations of the VSE include the simplified assumptions in the Mixed-Integer Quadratic Programming (MIQP) model.

Franssen (2022) conducted a comparative study to analyze the performance of different neural networks in predicting Formula 1 racing outcomes for the 2021 season. After preprocessing, both networks were trained using categorical cross entropy loss and the Adam optimizer. The Deep Neural Network (DNN) and Radial Basis Function Neural Network (RBFNN) significantly outperformed other models with the DNN achieving a slightly better F1 score of 58% over the 55% of the RBFNN. The DNN also exhibited better generalization than the RBFNN. Outside motorsport, transformer architectures have achieved strong results for financial time-series price/direction forecasting (Gezici and Sefer, 2024).

Piccinotti et al. (2021) proposed the application of online planning algorithms for race strategy optimization in Formula 1. Due to the task being a sequential decision making problem, the authors proposed Q-learning Open-Loop Upper Confidence Tree (QL OL-UCT), an agent which combines Monte Carlo sampling with Temporal Difference (TD) in order to tackle the high variance. The hyperparameters were tuned using Bayesian optimization. It was tested using historical race data from the years 2015–2018 and the results proved that it outperformed all existing works. The authors acknowledged that since the reward function focused on minimizing lap time, it sometimes led to impossible overtakes. The authors also concluded that open-loop online planning has a huge scope for race strategy optimization in motorsports. Complementary to RL approaches, hybrid Transformer–CNN designs report competitive direction-prediction accuracy on noisy financial sequences, underscoring the value of attention for sequential signals (Tuncer et al., 2022).

Sicoie (2022) developed a machine learning framework in order to predict the Formula 1 race winners and the final championship standings by leveraging ensemble regressors and support vector methods. Using historical data from the years 2014–2020, three models namely, Random Forest Regressor (RFR), Gradient Boosting Regressor (GBR), and Support Vector Regressor (SVR) were trained. Preprocessing included feature engineering by using domain knowledge and the model tuning was performed through RandomizedSearchCV. Race level predictions demonstrated low accuracy with the SVR achieving a R-squared score of 40.4%. However, the cumulative season standings predictions were more promising as the GBR achieved the highest Spearman rank correlation at 90.3%, followed by RFR at 90.2% and SVR at 88.3%. The author mentions that on analysis of feature importance, heavy reliance on driver and the constructor was identified. This was validated as it favored Lewis Hamilton (driver) and Mercedes (constructor) due to historical dominance.

Rondelli (2022) analyzed the use of deep learning models in order to predict optimal tire strategies in Formula 1 races. Telemetry data obtained from the FastF1 API was utilized for the same. The study compared three neural architectures: Long Short Term Memory (LSTM), Gated Recurrent Unit (GRU), and a standard Multi Layer Perceptron (MLP). A blind classifier which predicts the class labels solely through prior probabilities achieved an accuracy of 24.5%. The GRU proved to be the most effective model as it attained an accuracy of 51.4% with a loss 11.8% over LSTM which achieved an accuracy of 23.8% and loss of 16%.

Thomas et al. (2025) proposed an explainable reinforcement learning framework by collaborating with the Mercedes-AMG PETRONAS Formula 1 team, which has won nine Constructors' Championships and Imperial College, London. A neural policy was developed using Proximal Policy Optimization (PPO) in order to maximize the rewards based on race outcomes such as finishing position and time. The trained reinforcement learning agent showcased an impressive 8.6 second average improvement in comparison to the fixed strategies and it also ranked among the top five on the grid for about 76% of the races. Notably, the strategy selection was open loop which meant that no decisions were made after the race began. SHAP values were leveraged in order to examine the feature importance.

Aguad and Thraves (2023) developed a framework for optimizing pit stop decisions in Formula 1 by modeling it as a zero sum feedback Stackelberg game. In this game-theoretic approach, one of the teams (considered the leader) chose a particular strategy and then the opponent team (considered the follower) strives to optimize their response in real time. On analysis, this approach yielded an average race time improvement of about 2.3 seconds. The model also reduced the probability of being undercut by about 17.8%. The strength of the model lies in its ability to adapt to certain adverse race conditions including extreme wet weather and deployment of the safety car. A case study on the Italian Grand Prix that took place in Monza during 2021 revealed that the proposed framework could have altered the pit stop strategy of the McLaren F1 team and in turn would have disrupted the podium order.

García Tejada (2023) studied the application of machine learning algorithms in order to optimize the pit stop timing in Formula 1. The study utilized race data from the years 2019–2022 and applied three different models: Random Forest (RF), Support Vector Machines (SVM), and Artificial Neural Networks (ANN). The two binary target variables were: “has pit stop”, which indicated the occurrence of a pit stop and “good pit stop” which analyzed the effectiveness of the pit stop based on the change in track position. SVM proved to be the most effective model by outperforming the others in both variables by achieving an F1 score of 0.437 for “good pit stop” and 0.621 for “has pit stop”. The author concluded that machine learning algorithms can be used as decision-support tools rather than completely replacing human expertise.

In Daly (2023), the author analyzed the impact of meteorological conditions on Formula 1 races using machine learning techniques. The author constituted a dataset containing 348 races from the years 2005–2022. Ergast Developer API was used to extract race related features such as lap times and track position. The environmental features such as temperature and wind speed were obtained from Visual Crossing. Multiple standard Python libraries were utilized to perform data pre-processing and feature engineering. Variance Inflation Factor (VIF) analysis helped in handling high multicollinearity among features. Five regression models: Linear Regression, Ridge, Lasso, Random Forest, and Random Forest along with Grid Search were leveraged in order to predict multiple target variables. Upon analysis, the grid search optimized Random Forest outperformed all other models by attaining an R-squared score of 38.52% while the worst performing model was Lasso with an R-squared score of just 2.4%. The author concluded that the influence of weather on race outcomes proved to be statistically significant.

In Cheteles (2024), the author analyzed the prediction of Formula 1 race standings by contrasting feature importance with feature selection techniques. A dataset was constructed and contained features such as average braking points and grid positions. Domain specific transformations were performed in order to engineer additional features in order to improve model performance. Random Forest Regressor (RFR) and Gradient Boosting Regressor (GBR) models were deployed for predictions. Results proved that the model based on feature importance extracted the best performance by attaining a root mean square error (RMSE) of just 0.005 using RFR and 0.006 using GBR. In contrast, the feature selection based model exhibited higher deviations: 0.93 for RFR and 2.23 for GBR.

Fatima and Johrendt (2023) proposed Deep-Racing which is an Embedded Deep Neural Network (EDNN) model curated for predicting the optimal pit stop timings and final race positions in Formula 1. The study utilized data from about 169,000 laps during the years 2015–2022. The model combines two different EDNNs: one for predicting the position of the driver and another one for finding the optimal pit stop window. The dataset which was collected from multiple sources including Ergast API was subjected to pre-processing that included outlier filtering and feature imputation. All the features that possessed more than 85% pair-wise correlation were subsequently removed. The EDNN which predicted the driver ranks achieved an RMSE value of 2.05 and an R-squared value of 0.39 on the test set. Similarly, the model for the pit stop prediction demonstrated a precision of 0.56, recall of 0.83, and an overall F1 score of 0.67. Related work in market prediction also shows that attention-driven transfer learning over visual/sequence attributes can be effective (Pala and Sefer, 2024).

In the paper by Menon et al. (2024), the authors performed a data-driven analysis of historical Formula 1 telemetry data in order to uncover the proportional impact of driver skill against constructor performance on race outcomes. An extensive dataset from multiple sources was constituted and contained data which spanned over 70 years (1951–2021). The authors used a hybrid framework by leveraging linear regression modeling along with Monte Carlo simulations in order to forecast the race standings. The model resulted in a R-squared value of 0.88 which concluded that 88% of the variance in race outcomes can be traced back to the constructor. Therefore, in the modern era of Formula 1, the performance of the cars outweighed the skill set of the drivers. The authors also recommend using the model in multi-agent sports like MotoGP and WEC.

Pontin (2023) explored the potential of integrating artificial intelligence for optimization of race strategy in motorsports. The author proposed the development of an AI based race strategy assistant. Tire performance was evaluated based on the data collected from simulated driver in the loop (DIL) sessions. Upon extensive analysis, the author found that the 1-stop strategy (Soft–10 laps, Medium–17 laps), resulted in better lap times than a 2-stop strategy (Soft–10, Soft–10, Medium–13). The final component of the framework involved automating the race strategy execution via an artificial neural network (ANN). The dataset which the network was trained on suffered from high class imbalance due to which customized class weights were applied.

Class imbalance is a common challenge faced in classification tasks such as motorsport telemetry prediction. Class imbalance happens when few classes are underrepresented in comparison to other classes. In order to tackle the problem, Chawla et al. (2002) proposed the Synthetic Minority Over-sampling Technique (SMOTE). Instead of replicating the minority class instances, SMOTE creates synthetic samples by interpolating between existing minority class samples and their k-nearest neighbors. Leveraging SMOTE for pit stop window optimization might be beneficial as it ensures that the models are not biased toward dominant patterns.

**Figure 1:**
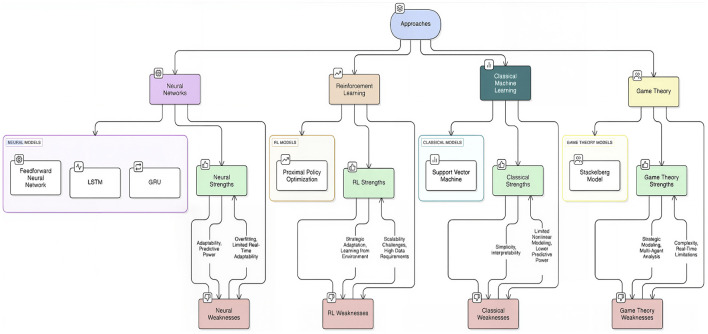
Overview of machine-learning approaches with key strengths and weaknesses for pit-stop strategy.

Despite extensive research into optimizing race strategies and anticipating pit stops for Formula 1, several gaps remain. A lot of the existing literature focuses on static or fixed strategies and tends to ignore dynamic real-time adaptations dependent on changing race conditions. Therefore, the development of an interpretable, adaptive, and context-aware pit stop prediction framework is urgently needed that robustly copes with class imbalance, utilizes multimodal telemetry, and generalizes well across seasons and circuits. Figure 1 provides a comprehensive overview of these machine learning paradigms, highlighting their key strengths and weaknesses in the context of pit-stop strategy.

## 3 Background

A modern Formula 1 car can exceed 375 km/h in its bid to cross the checkered flag first. All teams operate under stringent technical, sporting, and financial regulations enforced by the Fédération Internationale de l'Automobile (FIA) (2024). Each season features more than twenty Grands Prix at iconic yet distinct circuits—including Silverstone (United Kingdom), Monza (Italy), and Marina Bay (Singapore)—with the lap count chosen so that the total race distance is approximately 305 km. tire strategy is a critical performance lever. The sole tire supplier, Pirelli, offers five dry-tire compounds (C1–C5, hardest to softest); three are nominated for each event and designated hard, medium, and soft. Track surface, ambient temperature, and expected degradation guide the selection, and under normal dry conditions every team must use at least two different dry compounds during the race.

### 3.1 Formula 1 pit stop dynamics

There is a huge team behind every lightning-quick pit stop that unfolds in Formula 1, generally consisting of around 23 members known as the pit crew members. Their roles vary, from tire gunners, who are in charge of operating the wheel gun to remove and fix the wheel nut, to the pit release coordinator, who gives the signal for the car to be released back on to the track after a successful pit stop. Upon receiving approval from the strategists, the race engineer instructs the driver to initiate standard pit stop protocols. Then, the driver has to confirm their approval by pressing the pit confirm button, which is present on the steering wheel. Pressing the pit button limits the maximum speed of the car to 80 km/h consistent with the regulations of the FIA. The drivers are then required to carefully align their cars within the designated pit box for their team. Subsequently, the car is lifted with the help of hydraulics to replace the old tires. Aerodynamic adjustments to the front and rear wing might also be performed by the pit crew if necessary. While other motorsports such as IndyCar and NASCAR allow for refueling of the cars during pit stops, Formula 1 banned it in 2010, citing safety concerns. Once the tires are secured, the gantry light turns green, which is a go-ahead signal for the driver. Then, the driver proceeds to exit the pit lane to go back to the race track. The FIA mandates the use of at least two different tire compounds during each dry weather race, which means that a team must perform at least one mandatory pit stop. In the modern day, a Formula 1 pit crew takes, on average, anywhere from two to five seconds for a pit stop with the record being achieved by McLaren, which took 1.80 seconds to service Lando Norris' car during the 2023 Qatar Grand Prix. Pit stops can also be utilized to serve penalties for race regulation violations, and the pit crew is refrained from performing any activity on the car during this time.

### 3.2 Types of pit stop strategies

Multiple technical factors have an impact on the pit stop strategy of the teams. One among those is the concept of “clean air” which refers to undisturbed flow of air over the aerodynamical portions of the car. The strategists in the pit-wall always try to send their drivers on track into a zone of clean air after a pit stop to gain maximum advantage over their rivals. However, “dirty air” decreases the effectiveness of the front wing of the car and leads to under-steer issues. Thermal degradation is another important factor as drivers tend to lose grip if their tires are hotter than the optimal tire temperature range. Also, front and rear tire balance is crucial as each track might stress either of them excessively than the other. The race engineers also keep a keen eye on the brake wear data and g-force telemetry to evaluate tire degradation to decide the optimal pit stop window. The two most common pit stop strategies that are used by modern Formula 1 teams are the “Undercut” and “Over-cut”. A driver undercuts the rival when they pit earlier than expected in order to gain an advantage by leveraging a fresher set of tires with no wear. In contrast, a driver over-cuts the rival by delaying the pit stop to gain track position by taking advantage of the clean air in the circuit while the rival pits for newer tires.

### 3.3 Interventions by race control during the race

The Race Director, who is a part of the Race Control, is responsible for conducting the race in adherence to the FIA regulations and also ensures that the race proceeds in a safe manner. Race Officials, also called Marshals are stationed around the circuit to ensure that the track is devoid of any debris, and also recover and clean up the crashed cars from the track. More importantly, they communicate with the drivers through a few standardized flags to warn them of potential hazards on the track. A few of the significant flags include, the Yellow Flag, which is waved to signal danger on the track. Once it is waved, drivers are expected to slow down and are prevented from overtaking other cars. In a few cases, a double yellow flag might be waved in order to signify a serious accident. When a red flag is shown, it indicates a temporary suspension of the session due to extreme weather conditions or a track blockage due to a very serious accident. Under a red flag, the cars must significantly slow down and enter the pit lane until Race Control deems it safe to race again.

Generally, these flags are accompanied by the deployment of a Safety Car or a Virtual Safety Car. The Safety Car is a special type of car deployed on the track to slow down the other cars on the track. All the drivers need to follow the Safety Car in a single-file formation until the track is cleared. The Safety Car acts as the pilot car at the front, while all other drivers are prevented from overtaking it. The race can resume once the Safety Car returns back into the pit lane. Alternatively, instead of deploying a physical car on the track for minor incidents, the Virtual Safety Car is deployed. The term “VSC” is displayed on the FIA light panels to indicate its deployment. Drivers are then required to reduce their speeds by 40% and maintain a specific time between the cars in front of them. Interestingly, the teams have an opportunity for a strategic masterstroke during these interventions, as pitting under the yellow or red flag minimizes the time loss when compared to pitting during the regular course of the race, since all the cars are moving at a significantly slower pace.

## 4 Methodology

### 4.1 Factors affecting pit stop strategy

**Figure 2:**
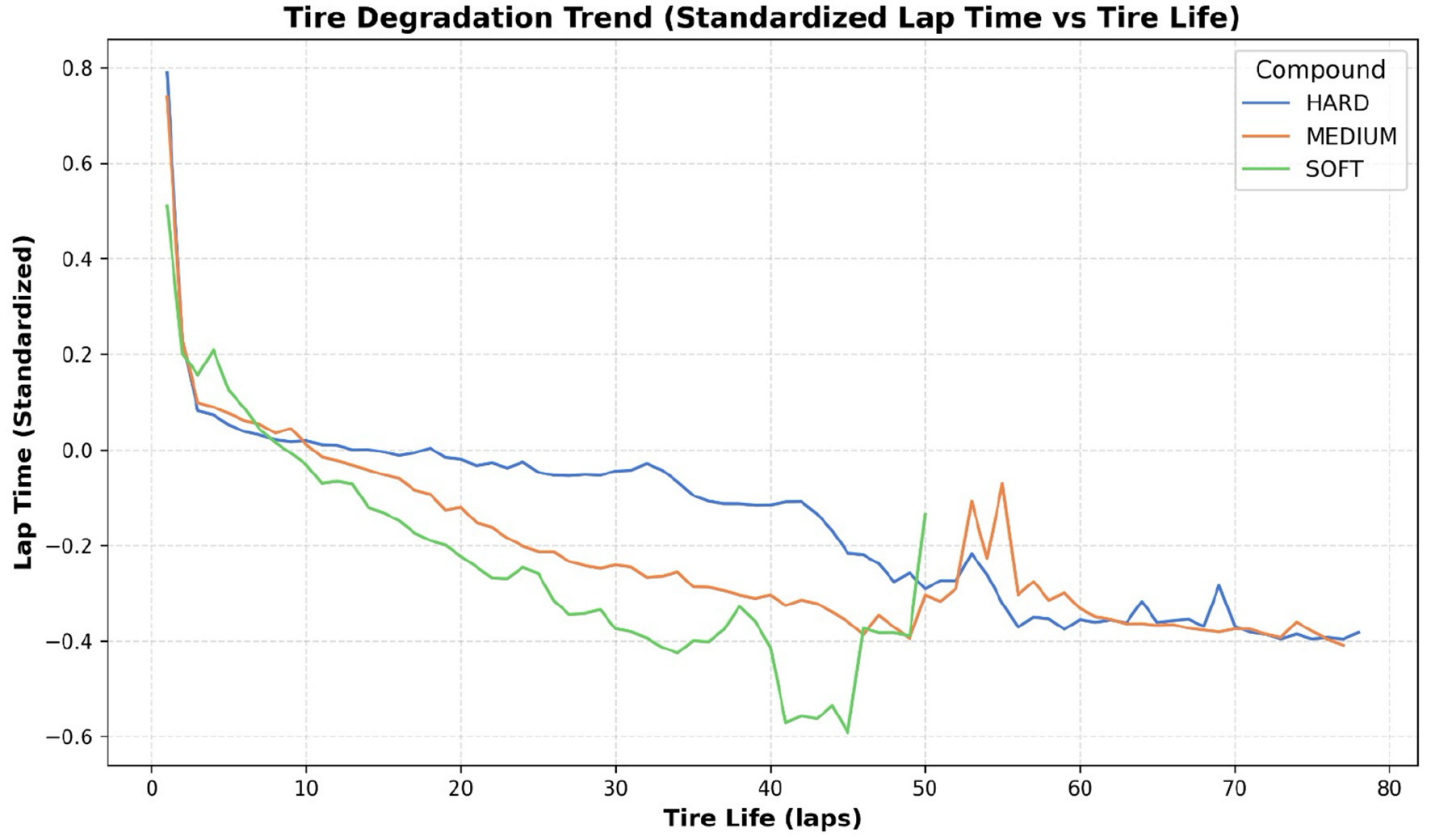
Tire degradation trend showing the standardized lap time against tire life for HARD, MEDIUM, and SOFT compounds. All compounds exhibit significant performance improvement initially, followed by gradual degradation, with softer compounds degrading more rapidly.

**Figure 3:**
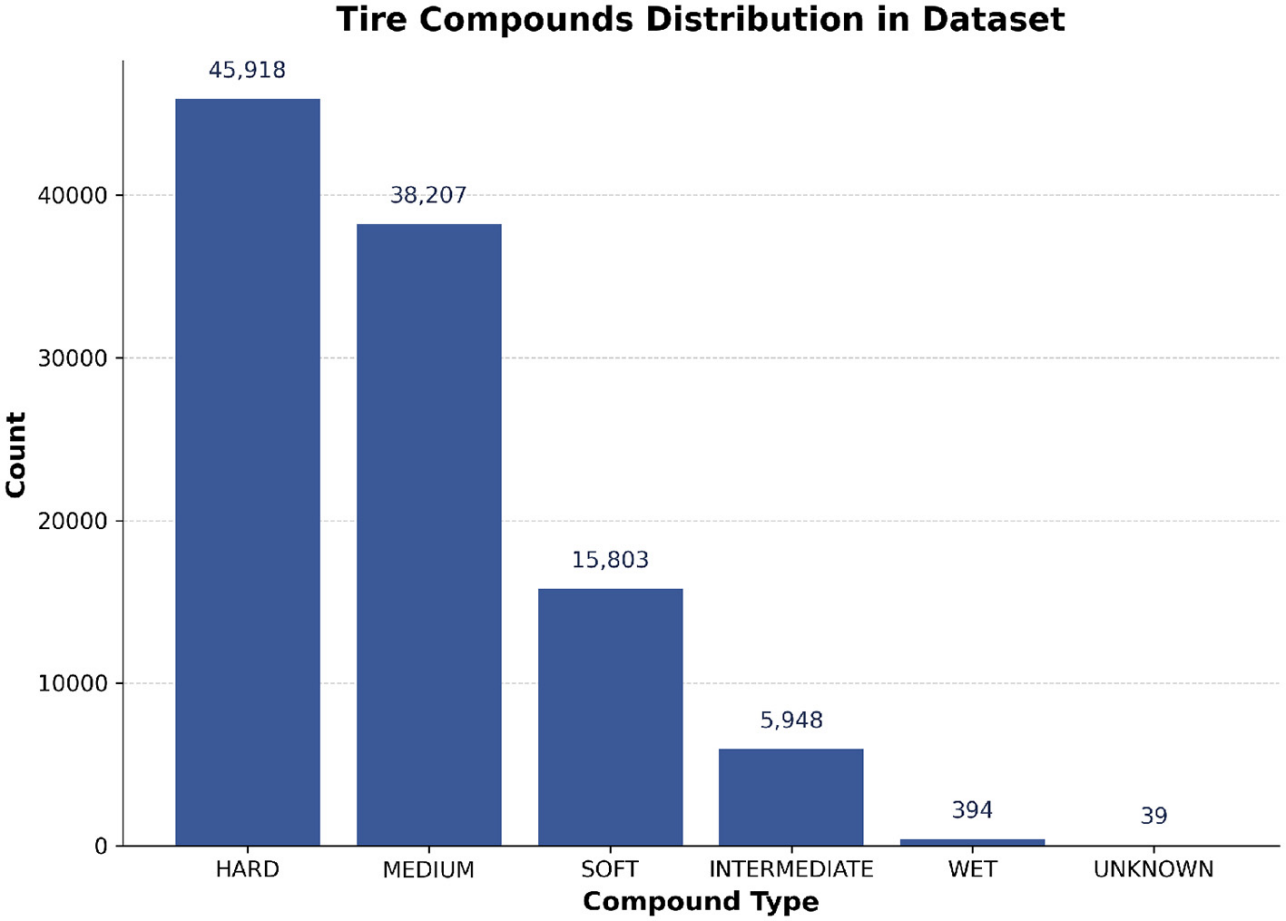
Distribution of tire compound types used in the dataset. The majority of laps were driven on hard and medium compounds, with softer or wet compounds being less frequent.

1. Driver and Driver Number: There always exists a skill gap between drivers, as each of them manage their tires differently.
2. Team: The set of strategists and race engineers in each team are essential for communication between the team and the driver on track. They receive and analyze valuable feedback from the driver and instruct the driver to follow the desired strategy.
3. Track Position: The position of the driver in the race plays a huge role in deciding the tire strategy and hence is a major factor in deciding the pit stop timing.
4. Lap Number: The remaining number of laps is crucial in deciding the optimal tire strategy.
5. tire Stint: Indicates the number of different tire compounds used by the driver during the race. The stint number remains the same until the driver pits and increases after they pit. In F1, a stint is the duration a driver stays on a particular tire compound before making a pit stop to switch to a new one.
6. tire Life: It represents the number of completed laps with a specific tire compound and helps to understand the trends in tire degradation, as shown in Figure 2.
7. Track Status: Indicates whether there are any hazardous incidents on track. Teams generally tend to take advantage of these situations, to reduce the time lost while performing their pit stops.
8. tire Compound: Since each tire compound provides varying grip levels and undergoes different levels of degradation, teams are required to make the right strategy call keeping the race situation in mind. As shown in Figure 3, the tire compound usage pattern indicates a strategic bias in compound selection throughout the race.
9. Event Name: Represents the location of the race track. It is a highly important parameter as each track location is different from the other and tailoring the strategy according to the specific track provides a competitive edge.
10. Laptime (in seconds): Monitoring the lap times of drivers is critical to observe any irregularities in tire degradation. Significant decrease in lap times might force teams to pit their drivers earlier than planned.

### 4.2 Data preprocessing

The dataset for this research was constructed by programmatically querying the FastF1 Python library. FastF1 is an open-source tool that serves as an API wrapper for accessing and parsing the rich, high-frequency data generated during F1 race weekends. It sources its information directly from the official Formula 1 live-timing data feeds, ensuring a high degree of accuracy and reliability. Unlike static, pre-compiled datasets, FastF1 provides the flexibility to extract a wide array of synchronized data streams, which was essential for building the feature set for our deep learning models. A function get_races(year) is defined in order to return a DataFrame that contains data about both the lap times and the weather. All data rows containing “intermediate”, “wet” or “unknown” tires were dropped. The rationale for this decision is that pit stop strategies under dry conditions (using hard, medium, or soft tires) are primarily driven by predictable factors like tire degradation, stint length, and track position, which are ideal for modeling. In contrast, pit stops involving wet or intermediate tires are almost entirely reactive to sudden and unpredictable weather changes. In these scenarios, the decision to pit is dictated by the immediate wetness of the track and driver feedback, not by the long-term strategic patterns our models are designed to learn. Including these laps would introduce significant noise and unpredictability, undermining the model's ability to learn the underlying patterns of strategic pit stops. The distribution shown in Figure 3 confirms that these tire types represent a small minority of the overall laps. Attributes such as AirTemp and Humidity were omitted due to their low significance in predicting the target attribute.

The attributes Time and LapTime were converted into seconds to enable numerical computations. The final eight races of 2024 were utilized for testing, whereas the others were considered as training data. A thorough data integrity analysis was conducted prior to model training, revealing a limited amount of missing data across three key attributes. The continuous variables, LapTime_Seconds and Position, contained 1,675 and 117 missing values, respectively. Having a total dataset size of 99,928 laps, these figures represent 1.68% and 0.12% of the data for each attribute respectively, likely resulting from sensor transmission errors or invalidated laps. Additionally, the categorical Track_Status column, which indicates race conditions such as “Yellow Flag” or “Safety Car”, contained 23 null values, representing a negligible 0.02% of the data. Given that the vast majority of a race is conducted under normal, “Clear” conditions (Track Status “1”), these null values were imputed using the mode of the column.

For the continuous Position and LapTime_Seconds attributes, a two-stage preprocessing pipeline was implemented to handle missing values and normalize the data. First, both attributes were normalized using StandardScaler. This initial step was critical because the chosen imputation method, K-Nearest Neighbors, is a distance-based algorithm. Normalization rescales the features to have a mean of 0 and a standard deviation of 1, ensuring that features with vastly different scales (e.g., lap times in seconds vs. track position from 1 to 20) contribute equally to the distance metric used for finding the “nearest neighbors”. Without this step, the feature with the larger scale would disproportionately influence the imputation. Following normalization, the missing values were imputed using the K-Nearest Neighbors (KNN) Imputer with a hyperparameter of k = 5. This method was chosen for its ability to provide a contextually aware estimate by analyzing the five most similar laps in the normalized feature space. This multivariate approach preserves the complex relationships within the data far more effectively than simpler methods like mean imputation. This entire process was designed to prepare the data for model training without deleting any rows, which would have disrupted the sequential integrity of the time-series data essential for our recurrent models.

**Figure 4:**
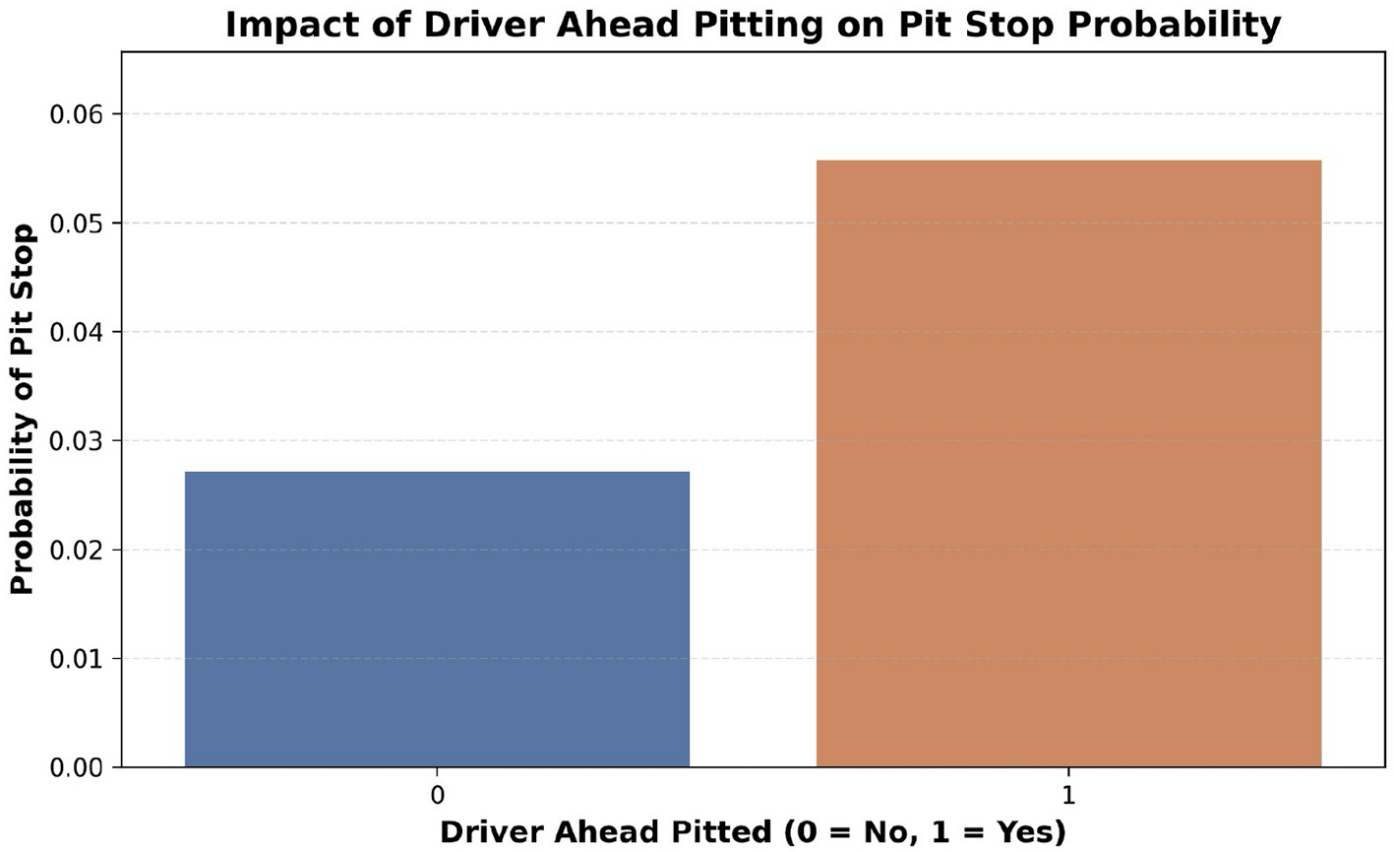
Impact of the preceding driver's pit decision on pit stop probability. The likelihood of a driver pitting increases significantly when the driver immediately ahead has already pitted, indicating strategic influence and team coordination in pit stop decisions.

The attribute CumulativeTimeStint was added in order to track the time taken by drivers in each of their stints which enabled a better understanding of the tire wear. Subsequently, the attributes DriverAheadPit and DriverBehindPit were added to indicate whether the drivers in front and the back have pitted. Figure 4 illustrates the impact of the DriverAheadPit attribute, highlighting its influence on pit stop decisions based on the behavior of preceding drivers. Stint changes were taken into consideration to avoid incorrect pit stop tracking. Finally, the data was sorted to ensure sequential ordering before proceeding with model building.

**Figure 5:**
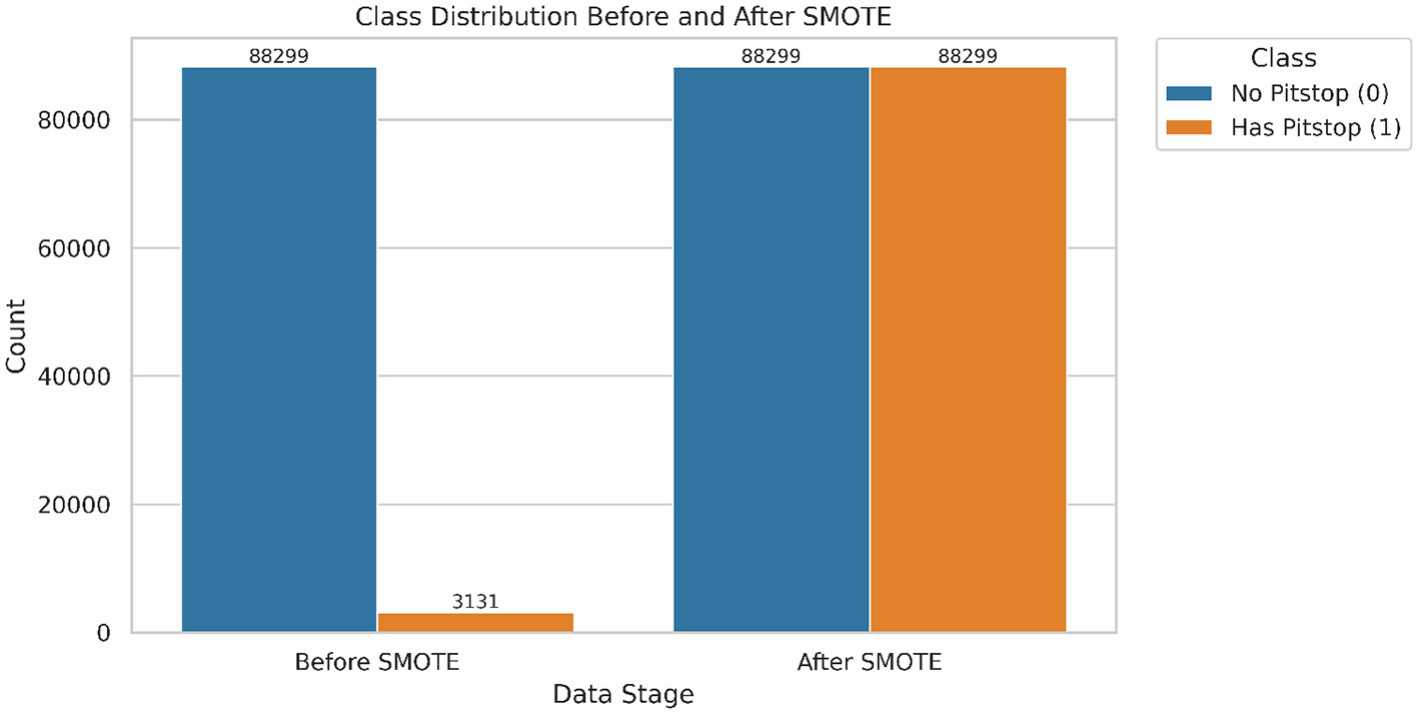
Class distribution before and after applying SMOTE. The minority class (*Has Pitstop* = *1*) is synthetically balanced with the majority class to address the data imbalance and improve generalization.

The addition of attributes, namely delta_laptime and race_progress_fraction proved to be crucial for analysis. delta_laptime indicated the difference in lap time when compared to the previous lap. race_progress_fraction indicated the current progress of the driver in the race by taking into the account the total number of laps. One-Hot Encoder was leveraged in order to convert the categorical columns Compound, Driver, Team, and EventName into binary. The target attribute HasPitStop contained binary values in which, one represents a pit made by the driver and zero represents no pit stop. As illustrated in Figure 5, the original dataset exhibited a significant class imbalance. Before resampling, the dataset contained 88,299 non-pit stop instances (class 0) and only about 3131 pit stop instances (class 1). This represents a split where pit stop laps account for less than 3.5% of the total data. To address this imbalance, which could bias the model toward the majority class, we applied the Synthetic Minority Over-sampling Technique (SMOTE). After SMOTE, the minority class (HasPitStop = 1) was up-sampled to match the majority class, resulting in a perfectly balanced dataset of 88,299 instances for each class, ready for model training.

### 4.3 Proposed deep learning models

#### 4.3.1 Bi-LSTM

Long Short-Term Memory (LSTM) networks are a special type of Recurrent Neural Networks (RNNs) which are designed in order to eliminate the vanishing gradient problem while storing long term dependencies. Unlike other RNNs which fail in retaining historical patterns, LSTMs are effective in capturing the long term dependencies as they leverage gates that control information transfer. The gates allow the network to either selectively retain or selectively forget. Information loss associated with uni-directional processing of data is prevalent in LSTMs. To evade this problem, Bidirectional LSTMs (Bi-LSTM) can be leveraged as it processes the information in both forward and backward direction. It provides a huge advantage as the model is able to study both from the past and the future time steps. This dual nature offers improved predictive power when compared to traditional models.

**Figure 6:**
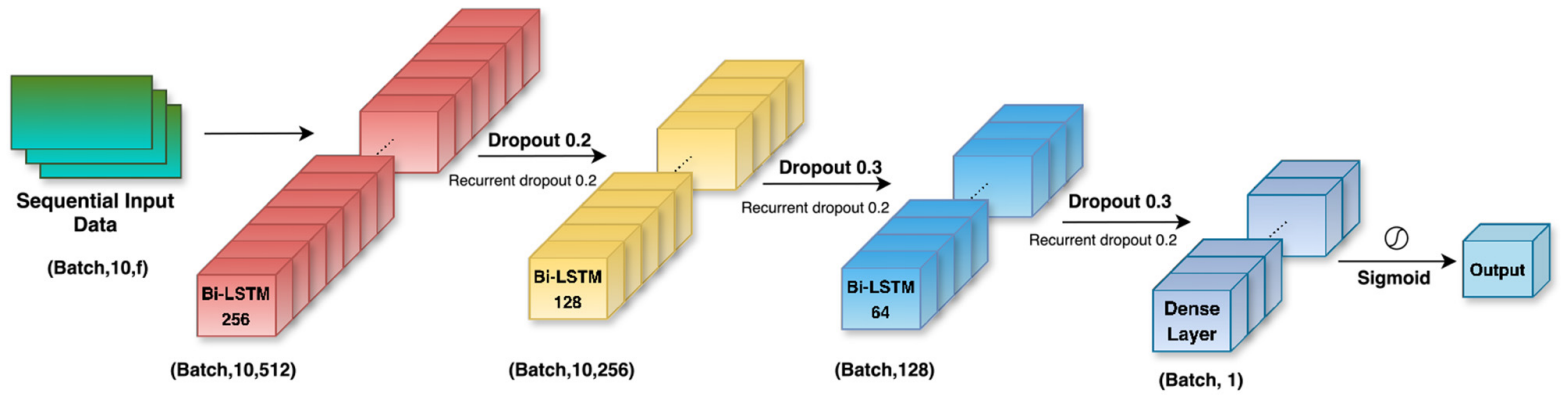
Architecture of the proposed Bi-LSTM model. The network consists of three stacked Bi-LSTM layers with decreasing hidden units (256, 128, 64), followed by a dense layer and a sigmoid activation for binary classification. Dropout and recurrent dropout are applied after each recurrent layer to enhance generalization and reduce overfitting.

Bi-LSTMs are equipped to handle the dynamic nature of the telemetry data of motorsports such as Formula 1. The study uses a Bi-LSTM model with 3 layers (256, 128, and 64 LSTM units respectively) along with a fully connected dense layer which contains a sigmoid activation function. The overall 3D architecture of the Bi-LSTM model, including its layered structure and flow of data through the network, is illustrated in Figure 6. In order to optimize convergence, training was performed with early stopping and learning rate reduction. Additionally, an optimal classification threshold was computed from the precision recall curve which enabled better F1-score alignment.

Every LSTM unit contains the following four gates:

- **Input Gate** (*i*_*t*_) - This gate decides the information that needs to be stored.
- **Forget Gate** (*f*_*t*_) - This gate decides the amount of past information that needs to be forgotten.
- **Cell Candidate** ($\tilde{c_{t}}$) - Contains the information about the new candidate.
- **Output Gate** (*o*_*t*_) - Controls the amount of exposure of the cell state as output.

The mathematical representations include:

- Input gate: Determines the influence of new input on the memory (Hochreiter and Schmidhuber, 1997).

(1) $$\begin{array}{l} i_{t}=\sigma \left(W_{i}x_{t}+U_{i}h_{t-1}+b_{i}\right) \end{array}$$

- Forget gate: Controls the retention of past memory content.

(2) $$\begin{array}{l} f_{t}=\sigma \left(W_{f}x_{t}+U_{f}h_{t-1}+b_{f}\right) \end{array}$$

- Candidate cell state: Proposes new content to be added to the memory.

(3) $$\begin{array}{l} \tilde{c}t=\tanh\left(W_{c}x_{t}+U_{c}ht-1+b_{c}\right) \end{array}$$

- Cell state update: Combines past and candidate content for memory update.

(4) $$\begin{array}{l} c_{t}=f_{t}⊙c_{t-1}+i_{t}⊙{\tilde{c}}_{t} \end{array}$$

- Output gate: Regulates how much memory affects the current output.

(5) $$\begin{array}{l} o_{t}=\sigma \left(W_{o}x_{t}+U_{o}h_{t-1}+b_{o}\right) \end{array}$$

- Hidden state: Final output of the cell for the current time step.

(6) $$\begin{array}{l} h_{t}=o_{t}⊙\tanh\left(c_{t}\right) \end{array}$$

- Bidirectional output: Concatenation of forward and backward hidden states (Graves et al., 2013).

(7) $$\begin{array}{l} h_{t}^{bi}=\text{concat}\left(h_{t}^{\text{forward}},h_{t}^{\text{backward}}\right) \end{array}$$

The variables used in the above equations describe the internal mechanisms of a Bi-LSTM network. At each time step *t*, the input vector *x*_*t*_ consists of relevant race telemetry features such as lap time, tire compound, and stint number. The hidden state *h*_*t*_ represents the short-term memory that is updated at every time step, while the cell state *c*_*t*_ stores long-term information across the sequence. The input gate *i*_*t*_ determines how much of the new candidate value $\tilde{c}t$ should be added to the cell memory. The forget gate *f*_*t*_ decides which parts of the previous cell state *c*_*t*−1_ should be retained. The output gate *o*_*t*_ controls the amount of memory that is passed to the hidden state. Weight matrices *W* and *U* are trainable parameters that apply transformations to the current input and previous hidden state, respectively, while b_*_ represents the bias terms. The functions σ(·) and tanh(·) are used to introduce non-linearity. Element-wise multiplication is denoted by ⊙. Finally, $h_{t}^{\text{bi}}$ is the concatenated output from both the forward and backward LSTM passes, enabling the model to utilize full temporal context from both directions. The optimized hyperparameter configuration used for training the Bi-LSTM model is shown in Table 1.

**Table 1:** Hyperparameter settings for BiLSTM model.

**Hyperparameter**	**Value**
Sequence length (timesteps)	10
Number of layers	3 Bidirectional LSTM layers
LSTM units	256 → 128 → 64
Dropout rates	0.2, 0.3, 0.3
Recurrent DROPOUT RAtes	0.2, 0.2, 0.2
Optimizer	Adam
Learning rate	5 × 10^−4^
Loss function	Binary Crossentropy
Batch size	32
Epochs	50 (with Early Stopping)
Early stopping patience	5 epochs
ReduceLROnPlateau patience	3 epochs
Learning rate reduction factor	0.5
Minimum learning rate	1 × 10^−6^
Class weights	{0: 1.0, 1: 3.0}
Oversampling strategy	SMOTE (*sampling_strategy* = 1.0)
Validation split	20% (Stratified)

**Figure 7:**
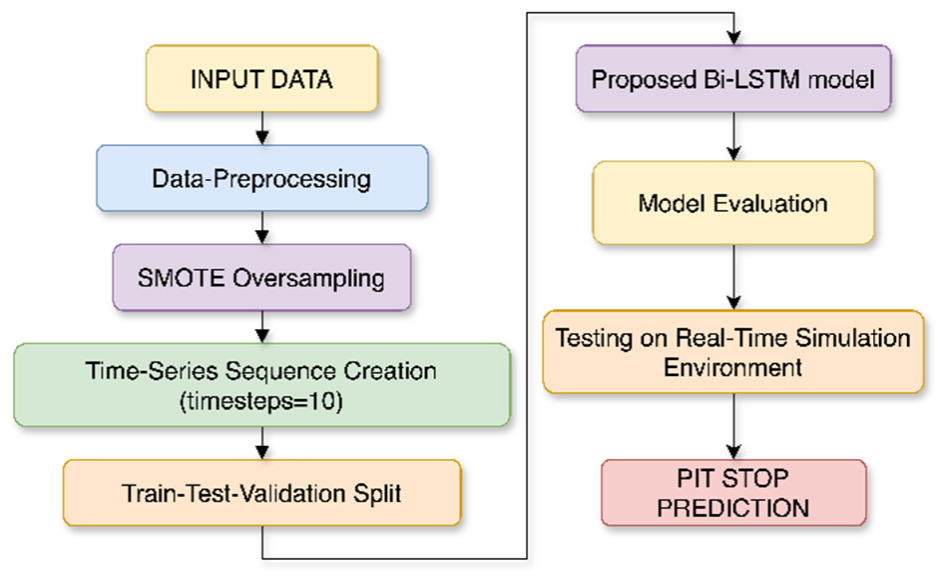
Workflow of the proposed pit stop prediction model using Bi-LSTM. The pipeline includes preprocessing, SMOTE-based oversampling, time-series conversion, training, and simulation by visualizing the race event.

Figure 7 illustrates the complete workflow of the proposed pit stop prediction model, showcasing each stage from raw telemetry preprocessing and SMOTE-based oversampling to Bi-LSTM model training, testing, and validation using a historical race visualization interface. This race visualization interface is created using the Drivers position data and Track data for each event, which are obtained from the same FastF1 library to visually simulate the actual race movements and position changes of all drivers throughout the race.

#### 4.3.2 TCN–GRU

The specialty of the TCN-GRU model is that it brings together the Temporal Convolutional Networks (TCN) with Gated Recurrent Units (GRU). By doing this, it combines both recurrent and convolutional architectures in order to perform sequential data modeling. Stable training is guaranteed by the residual connections as they enhance the gradient flow while the TCN portion effectively captures the long range dependencies. Subsequently, the GRU layer is crucial for selectively retaining the required sequence data. This approach also eliminates the disadvantages of traditional recurrent models such as very slow training times and short term dependency biases. Additionally, including dropout regularization and stratified validation reduces the risk of overfitting.

The mathematical representations of TCN-GRU include:

- Dilated causal convolution for extracting long-range patterns (Hewage et al., 2020):

(8) $$\begin{array}{l} y_{t}=\overset{k-1}{\underset{i=0}{\sum }}x_{t-d\cdot i}\cdot w_{i}+b \end{array}$$

- Update gate: controls balance between past state and new input (Yu et al., 2023):

(9) $$\begin{array}{l} z_{t}=\sigma \left(W_{z}x_{t}+U_{z}h_{t-1}+b_{z}\right) \end{array}$$

- Reset gate: determines influence of past hidden state:

(10) $$\begin{array}{l} r_{t}=\sigma \left(W_{r}x_{t}+U_{r}h_{t-1}+b_{r}\right) \end{array}$$

- Candidate hidden state: encodes new content:

(11) $$\begin{array}{l} \tilde{h}t=\tanh\left(W_{h}x_{t}+U_{h}\left(r_{t}⊙ht-1\right)+b_{h}\right) \end{array}$$

- Final hidden state: merges past memory and new candidate:

(12) $$\begin{array}{l} h_{t}=\left(1-z_{t}\right)⊙h_{t-1}+z_{t}⊙{\tilde{h}}_{t} \end{array}$$

- Binary cross-entropy loss function for optimization (Goodfellow et al., 2016):

(13) $$ℒ=-\frac{1}{N}\sum_{i=1}^{N}\left[y_{i}\log({\hat{y}}_{i})+(1-y_{i})\log(1-{\hat{y}}_{i})\right]$$

In this model, *x*_*t*_ denotes the input vector at time step *t*, while *d* and *k* represent the dilation factor and kernel size used in the convolutional layer to capture long-range dependencies. The weights *w*_*i*_ and bias *b* are learnable parameters of the TCN. In the GRU block, *z*_*t*_ and *r*_*t*_ are the update and reset gates, regulating how past information influences the current state. The hidden state *h*_*t*_, previous state *h*_*t*−1_, and candidate state $\tilde{h}t$ define the memory dynamics. *W* and *U* are weight matrices applied to the input and hidden layers, with b_*_ as biases. The functions σ and tanh are activation functions, and ⊙ indicates element-wise multiplication. The loss L computes the binary classification error over predictions ŷ_*i*_ and true labels *y*_*i*_.

#### 4.3.3 GRU

The Gated Recurrent Unit (GRU) is a more powerful adaptation of RNNs as it is designed in a manner that overcomes the limitations of traditional RNNs while being a lightweight alternative to LSTMs. The GRU would be an effective model as it is curated to analyze important temporal dependencies as in the case of pit stop prediction. The strength of the GRU model rests in the presence of gating mechanisms which control the flow of information and decide what to retain. GRUs also excel in handling imbalanced datasets in scenarios like pit stop prediction where the number of regular laps is significantly higher than the pit stop laps. The GRU-based model in this study shares the same gating mechanisms as detailed in Yu et al. (2023).

#### 4.3.4 InceptionTime

While the Inception models were originally designed for image processing predominantly, the InceptionTime model extends their use case to time series classification. The InceptionTime model leverages multiple convolutional branches with varying kernel sizes in order to capture patterns. The residual connections play a huge role in stabilizing training. The model also filters out irrelevant changes in the data. The InceptionTime model also uses Global Average Pooling (GAP) in place of fully connected layers to reduce overfitting. Since each of the F1 track venues are unique, InceptionTime model might come in handy as it has the ability to capture patterns at different scales.

The mathematical representations of InceptionTime include (Fawaz et al., 2020):

- 1D convolution for feature extraction over time windows:

(14) $$\begin{array}{l} y_{i}=\overset{k-1}{\underset{j=0}{\sum }}x_{i+j}w_{j}+b \end{array}$$

- Residual connection for stabilizing deep architecture:

(15) $$\begin{array}{l} F\left(x\right)=f\left(x\right)+x \end{array}$$

- Global average pooling across time steps for each feature map:

(16) $$\begin{array}{l} z_{c}=\frac{1}{T}\overset{T}{\underset{t=1}{\sum }}x_{t,c} \end{array}$$

In the equation for *y*_*i*_, the input sequence is denoted by *x*_*i*_, and the convolution filter weights are represented by *w*_*j*_, applied over a window of size *k*. The term *b* refers to the bias added after the convolution. The function *F*(*x*) = *f*(*x*)+*x* defines a residual connection, where *f*(*x*) is the output of a transformation applied to the input *x*, allowing the original input to be added directly to the result. In the final equation, *z*_*c*_ represents the average value computed over the time dimension *T* for each feature channel *c*, which is used in global average pooling to reduce the temporal dimension before classification.

#### 4.3.5 CNN-BiLSTM

The CNN-BiLSTM is a hybrid model that combines the spatial pattern gathering capabilities of the Convolutional Neural Networks (CNNs) along with sequential dependency capturing abilities of the Bidirectional Long Short-Term Memory networks (BiLSTM). The CNNs can be trained to capture the short-term dependencies in time-series inputs. Subsequently, the features are fed to the BiLSTM layers in order to be processed in both forward and backward directions. The strength of the model lies in its ability to capture both local variations (using the CNN layer) and long range trends (using BiLSTM layers). This dual capability is crucial for improving the model generalization.

The mathematical representations of CNN-BiLSTM include:

- 1D convolution to extract local patterns from input sequence (Zhang et al., 2023):

(17) $$\begin{array}{l} h_{t}=\text{ReLU}\left(W*x_{t:t+k-1}+b\right) \end{array}$$

- Max pooling to reduce dimensionality and retain dominant features (Zhang et al., 2023):

(18) $$\begin{array}{l} h_{t}^{pool}=\max\left(x_{t},x_{t+1},\ldots ,x_{t+p-1}\right) \end{array}$$

- Forward and backward LSTM passes to learn temporal dependencies (Hochreiter and Schmidhuber, 1997):

(19) $$\begin{array}{l} \vec{h_{t}}=\text{LSTM}\left(x_{t},\vec{h_{t-1}}\right),\;\overset{⃖}{h_{t}}=\text{LSTM}\left(x_{t},\overset{⃖}{h_{t+1}}\right) \end{array}$$

- Bidirectional hidden state concatenation (Graves et al., 2013):

(20) $$\begin{array}{l} h_{t}^{bi}=\left[\vec{h_{t}},\overset{⃖}{h_{t}}\right] \end{array}$$

- Final dense layer with sigmoid activation for binary prediction (Zhang et al., 2023):

(21) $$\begin{array}{l} \hat{y}=\frac{1}{1+e^{-\left(Wh+b\right)}} \end{array}$$

In these equations, *x*_*t*_ refers to the input feature vector at time step *t*, and *W*, *b* are the weight and bias of the convolution kernel. The operator * denotes 1D convolution, and ReLU is the activation function applied afterward. Max pooling $h_{t}^{pool}$ selects the highest value within a local region of the input. In the Bi-LSTM layer, $\vec{h_{t}}$ and $\overset{⃖}{h_{t}}$ are the forward and backward hidden states, which are concatenated to form $h_{t}^{bi}$. Finally, ŷ is the output probability produced by a sigmoid-activated dense layer for binary classification.

### 4.4 Model architecture, training, and hyperparameter configuration

To ensure a robust and transparent comparison, a systematic and consistent methodology was applied for the selection, configuration, and training of all deep learning models.

#### 4.4.1 Rationale for a data-driven deep learning approach

For this study, a purely data driven methodology using deep learning was selected over model based approaches, such as game theory or simulation based optimization, for three primary reasons. Firstly, deep learning models can learn complex, non-linear patterns directly from high dimensional telemetry data without requiring explicit and often simplified assumptions about the underlying race dynamics (e.g., tire degradation curves, opponent rationality) that are necessary for game-theoretic models. Secondly, the Formula 1 environment is highly non-stationary due to evolving regulations and car technologies. A data-driven model trained on recent data can adapt to these changes more fluidly than a formal model requiring recalibration. Finally, deep learning architectures are inherently scalable to the vast amount of time series data available, allowing them to capture subtle predictive signals that might be intractable to formalize in a traditional optimization framework. While game theory provides a powerful lens for strategic reasoning, our focus is on building a predictive support tool that learns directly from observed race behavior.

The five deep learning architectures were deliberately selected to represent a diverse and complementary set of state-of-the-art techniques for time-series classification. Each architecture possesses a unique inductive bias, making it well suited to capturing different types of patterns inherent in sequential Formula 1 telemetry data.

- Recurrent Architectures (Bi-LSTM and GRU): These models are the canonical choice for sequence modeling. The Bidirectional Long Short-Term Memory (Bi-LSTM) network was selected for its proven ability to learn long-range temporal dependencies. Its gated memory cells allow it to selectively retain information over long periods, which is essential for modeling cumulative effects like tire degradation. The Gated Recurrent Unit (GRU) was included as a strong, computationally efficient alternative.
- Hybrid Architectures (CNN-BiLSTM and TCN-GRU): These models were chosen to leverage the synergy between convolutional and recurrent layers. CNN-Bi-LSTM uses 1D convolutions to extract local motifs (e.g., sharp changes in lap time) before Bi-LSTM layers model their long-term sequential impact. The TCN-GRU uses a Temporal Convolutional Network with dilated convolutions to efficiently capture patterns across multiple time scales.
- State-of-the-Art Time-Series Classifier (InceptionTime): This architecture was included as a benchmark to evaluate our recurrent and hybrid models against a leading, non-recurrent architecture specifically designed for time-series classification. InceptionTime has demonstrated strong performance across numerous benchmark datasets, making it a suitable point of comparison.

#### 4.4.2 Model configuration and hyperparameter tuning

To ensure a scientifically valid and fair comparison, a highly controlled experimental setup was established. Each of the five architectures was individually optimized during a systematic, empirical tuning process. Although we experimented with different learning rates, class weights, and SMOTE strategies for each model, we found that the configuration presented here consistently yielded the best performance across the board. This finding allowed for a direct and unbiased comparison of their architectural merits, as performance differences could be confidently attributed to model structure rather than variations in the training protocol.

These shared training parameters, used for all five models, were as follows:

- **Optimizer:** Adam
- **Learning Rate:** 5 × 10^−4^
- **Loss Function:** Binary Crossentropy
- **Batch Size:** 32
- **Data Handling:** A *sampling_strategy* of 1.0 was used for SMOTE, supplemented by class weights of {0: 1.0, 1: 3.0} during training. A stratified 20% validation split was used for all models.

The key differentiators in our experiment were therefore the model architectures themselves. The final, optimized architecture for our proposed model, Bi-LSTM, is detailed in Table 1. The individually tuned architectures for the four comparative models are described below:

- **GRU:** Comprised of three stacked Bidirectional GRU layers with decreasing units (256 → 128 → 64), followed by two Dense layers (64 → 32).
- **TCN-GRU:** A hybrid model consisting of a single Temporal Convolutional Network block (64 filters, kernel size 3) followed by a single GRU layer with 64 units.
- **CNN-BiLSTM:** A hybrid model beginning with a 1D Convolutional layer (64 filters, kernel size 3) and a MaxPooling layer, followed by two stacked Bidirectional LSTM layers (128 → 64).
- **InceptionTime:** The standard architecture was used, with its internal depth and filter sizes determined through the empirical tuning process.

All models were designed to process input sequences with a length of 10 timesteps. To provide a more comprehensive evaluation, particularly given the class imbalance inherent in the dataset, model performance was assessed not only using precision, recall, and F1-score but also with Specificity, Balanced Accuracy, ROC-AUC (Area Under the Receiver Operating Characteristic Curve), and AUC-PR (Area Under the Precision-Recall Curve). This expanded set of metrics allows for a nuanced understanding of each model's ability to handle both the majority (no pit stop) and minority (pit stop) classes.

#### 4.4.3 Training protocol and convergence criteria

A standardized training protocol was used for all models to ensure a fair and controlled comparison, with the specific optimizer and loss function for each model detailed in Table 1. The training duration was capped at a maximum of 50 epochs. Actual convergence was managed dynamically using two Keras callbacks whose parameters, listed in the tables, were held constant across all experiments. Early Stopping served as the primary mechanism to prevent overfitting by halting the training if the monitored loss did not improve for a patience of five epochs. Concurrently, ReduceLROnPlateau facilitated finer-grained convergence by reducing the learning rate if the loss plateaued. As noted in the tables, the specific metric monitored (val_loss or loss) was tailored to whether a validation set was used for that particular model's optimal configuration.

#### 4.4.4 Evaluation metrics

To provide a robust and quantitative assessment of model performance, particularly given the significant class imbalance in the pit stop prediction task, a comprehensive set of evaluation metrics was employed. These metrics are derived from the confusion matrix, which categorizes predictions into four distinct outcomes:

- **True Positives (TP):** The number of laps correctly identified as a pit stop (*HasPitStop* = *1*).
- **True Negatives (TN):** The number of laps correctly identified as a non-pit stop (*HasPitStop* = *0*).
- **False Positives (FP):** The number of non-pit stop laps incorrectly classified as a pit stop (Type I error).
- **False Negatives (FN):** The number of pit stop laps incorrectly classified as a non-pit stop (Type II error).

Based on these components, the following metrics were calculated.

##### 4.4.4.1 Precision

Measures the accuracy of positive predictions. It answers the question: “*Of all the laps the model predicted as a pit stop, what fraction were actual pit stops?”*


(22) $$\begin{array}{l} \text{Precision}=\frac{TP}{TP+FP} \end{array}$$

##### 4.4.4.2 Recall (sensitivity or true positive rate)

Measures the model's ability to identify all actual positive instances. It answers the question: “*Of all the actual pit stops that occurred, what fraction did the model correctly identify?”*


(23) $$\begin{array}{l} \text{Recall}=\frac{TP}{TP+FN} \end{array}$$

##### 4.4.4.3 F1-score

The harmonic mean of Precision and Recall. It balances the trade-off between the two metrics, providing a single score that is particularly useful for imbalanced datasets.


(24) $$\begin{array}{l} \text{F1-Score}=\frac{2\times \text{Precision}\times \text{Recall}}{\text{Precision}+\text{Recall}}=\frac{2TP}{2TP+FP+FN} \end{array}$$

##### 4.4.4.4 Specificity (true negative rate)

Measures the model's ability to correctly identify all negative instances. It complements Recall.


(25) $$\begin{array}{l} \text{Specificity}=\frac{TN}{TN+FP} \end{array}$$

##### 4.4.4.5 Balanced accuracy

The arithmetic mean of Recall and Specificity. It is robust to class imbalance as it evaluates performance equally across both classes.


(26) $$\begin{array}{l} \text{Balanced Accuracy}=\frac{\text{Recall }+\text{ Specificity}}{2}\\ \;=\frac{1}{2}\left(\frac{TP}{TP+FN}+\frac{TN}{TN+FP}\right) \end{array}$$

##### 4.4.4.6 Area under the receiver operating characteristic curve (ROC-AUC)

Evaluates the model's discriminative ability across all classification thresholds. The ROC curve plots the True Positive Rate (Recall) against the False Positive Rate (1 - Specificity). An AUC of 1.0 indicates a perfect classifier, whereas an AUC of 0.5 reflects a model performing no better than random chance.

##### 4.4.4.7 Area under the precision–recall curve (AUC-PR)

Summarizes the trade-off between Precision and Recall across varying thresholds. It is often more informative than ROC-AUC when handling highly imbalanced datasets, as it emphasizes the performance of the minority class (*pit stops*).

#### 4.4.5 Computational environment

To ensure full reproducibility of our results, all experiments were conducted within a consistent and clearly defined computational environment.

- Hardware: All model training and evaluation were performed on a system equipped with a high-performance NVIDIA A100 GPU with 80 GB of VRAM. The host system included multiple virtual CPUs and 125 GB of system RAM.
- Software and Libraries: The experimental framework was built on Python (v3.9). The deep learning models were implemented using the TensorFlow (v2.11.0) framework with its integrated Keras API. Data manipulation, preprocessing, and evaluation were handled using the Pandas (v1.5.2), NumPy (v1.23.5), and Scikit-learn (v1.2.1) libraries. Class imbalance was addressed using the imblearn (v0.10.1) library. All final models and scalers were saved to disk using the native Keras .h5 format and joblib, respectively.

## 5 Results

**Figure 8:**
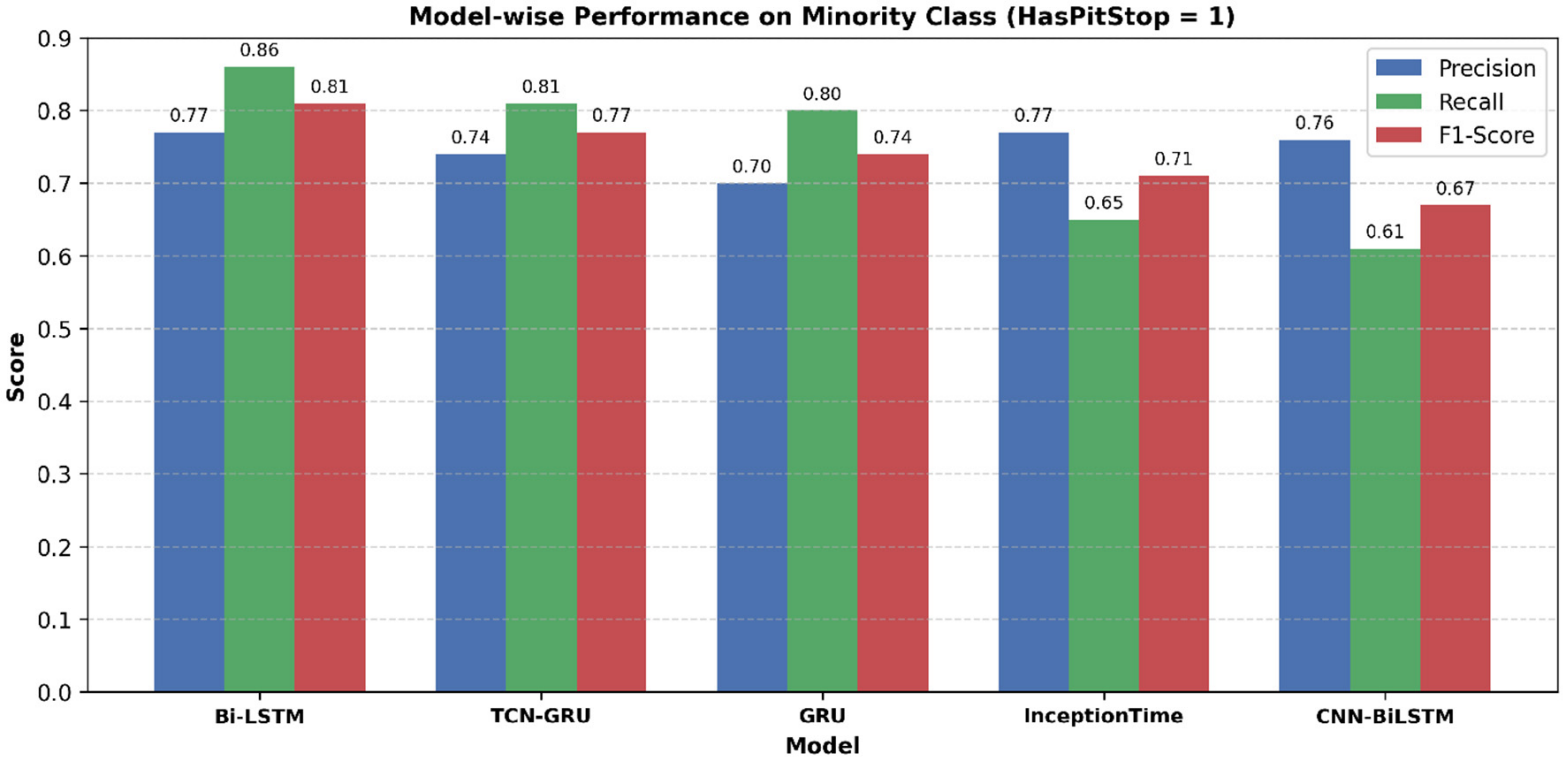
Model-wise comparison of precision, recall, and F1-score for the minority class (*HasPitStop* = *1*). Bi-LSTM achieves the highest F1-score, followed by TCN-GRU and GRU, indicating its robustness in pit stop prediction.

The study employed five deep learning models to comparatively analyze their performance on predicting pit stops in Formula 1 on the test set reported for the minority class (HasPitStop = 1). To account for the significant class imbalance in the test set, which has only 3% of pit stop occurrences, performance was analyzed using a comprehensive suite of metrics including Precision, Recall, F1-Score, Specificity, and Balanced Accuracy, as detailed in Table 2. The Bi-LSTM model demonstrated superior overall performance, achieving the highest F1-Score (0.81), Balanced Accuracy (0.93), and Recall (0.86), indicating its robust capability in correctly identifying the rare pit stop instances. The trade-offs between precision, recall, and the resulting F1-score for each model are visually summarized in Figure 8.

**Table 2:** Model-wise performance on minority class (*HasPitStop* = *1*).

**Model**	**Precision**	**Recall**	**F1-score**	**Specificity**	**Balanced accuracy**
Bi-LSTM	0.77	0.86	0.81	0.992	0.93
TCN-GRU	0.74	0.81	0.77	0.991	0.90
GRU	0.70	0.80	0.74	0.989	0.89
InceptionTime	0.77	0.65	0.71	0.994	0.82
CNN-BiLSTM	0.76	0.61	0.67	0.994	0.80

To rigorously validate our findings, we computed 95% confidence intervals for key performance metrics using 1,000 bootstrap resamples of the test set, with the comprehensive results summarized in Table 3. As shown in the table, the Bi-LSTM model not only achieved the highest F1-score (0.81) and Balanced Accuracy (0.926) but also exhibited tight confidence intervals, indicating stable and superior performance. Notably, the 95% confidence interval for the Bi-LSTM's F1-score [0.791, 0.832] does not overlap with those of the GRU, InceptionTime, or CNN-BiLSTM models. This pattern also holds for Balanced Accuracy. This provides strong evidence that the Bi-LSTM's superior performance is not due to random statistical variation. The confidence interval for the TCN-GRU model shows only a minor overlap, suggesting its performance is competitive and warrants direct statistical comparison.

**Table 3:** Model performance with 95% confidence intervals (CI).

**Model**	**F1-score**	**CI for F1-score**	**Balanced accuracy (BA)**	**CI for BA**
**Bi-LSTM**	**0.81**	**[0.791, 0.832]**	**0.926**	**[0.905, 0.941]**
TCN-GRU	0.77	[0.750, 0.795]	0.900	[0.881, 0.918]
GRU	0.74	[0.725, 0.770]	0.890	[0.839, 0.903]
InceptionTime	0.71	[0.682, 0.735]	0.824	[0.801, 0.849]
CNN-BiLSTM	0.67	[0.642, 0.698]	0.801	[0.776, 0.824]

Bold values indicate the best-performing model for the corresponding metric.

To formalize this comparison, we conducted pairwise McNemar's tests between the Bi-LSTM model and all other architectures. The resulting *p*-values are presented in Table 4. The results from the McNemar's tests confirm the conclusions drawn from the confidence intervals. At a significance level of α = 0.05, the Bi-LSTM model's predictive performance is statistically superior to all other models evaluated. The comparison with the TCN-GRU model yielded a *p*-value of 0.042, which, while significant, indicates a smaller performance margin compared to the other models. The highly significant *p*-values (< 0.001) in the comparisons against GRU, InceptionTime, and CNN-BiLSTM underscore a substantial and reliable performance gap.

**Table 4:** Pairwise statistical comparison of models using McNemar's test (*p*-values).

**Comparison**	***p*-value**	**Statistically significant? (α = 0.05)**
Bi-LSTM vs. TCN-GRU	0.042	Yes
Bi-LSTM vs. GRU	0.003	Yes
Bi-LSTM vs. InceptionTime	< 0.001	Yes
Bi-LSTM vs. CNN-BiLSTM	< 0.001	Yes

There are several reasons why the Bi-LSTM model performs significantly better than the baseline. First, its tendency to learn from temporal dependencies in both past and future directions makes it particularly powerful at modeling sequential lap data where decisions are influenced by developing trends including tire degradation, stint length, and track conditions. Furthermore, Bi-LSTM can handle sequences of variable length, which is consistent with the varying number of laps between pits across races and drivers.

**Figure 9:**
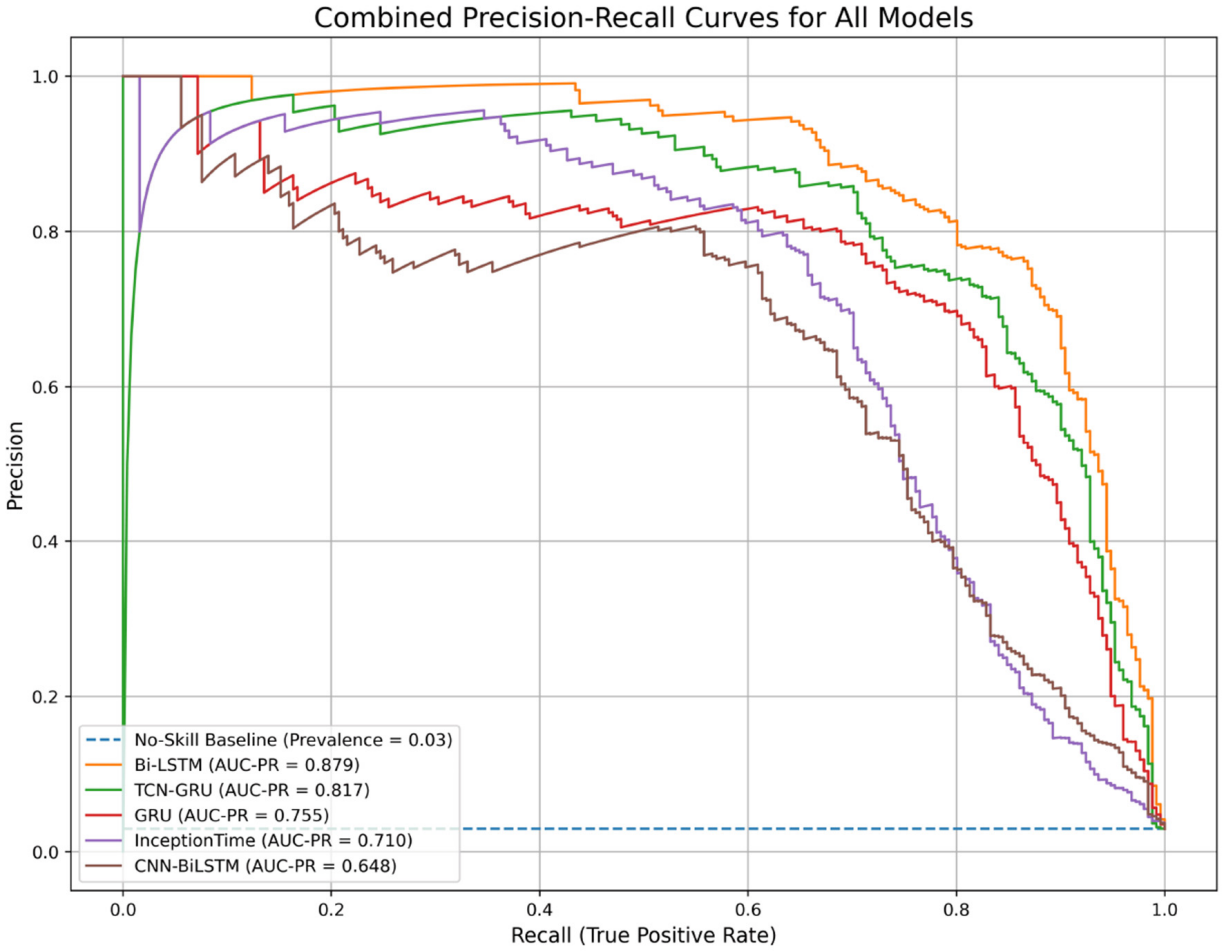
Combined Precision-Recall curves for all evaluated models. The area under the PR curve (AUC-PR) is a robust metric for imbalanced classification. The Bi-LSTM model demonstrates the best performance with the highest AUC-PR.

**Figure 10:**
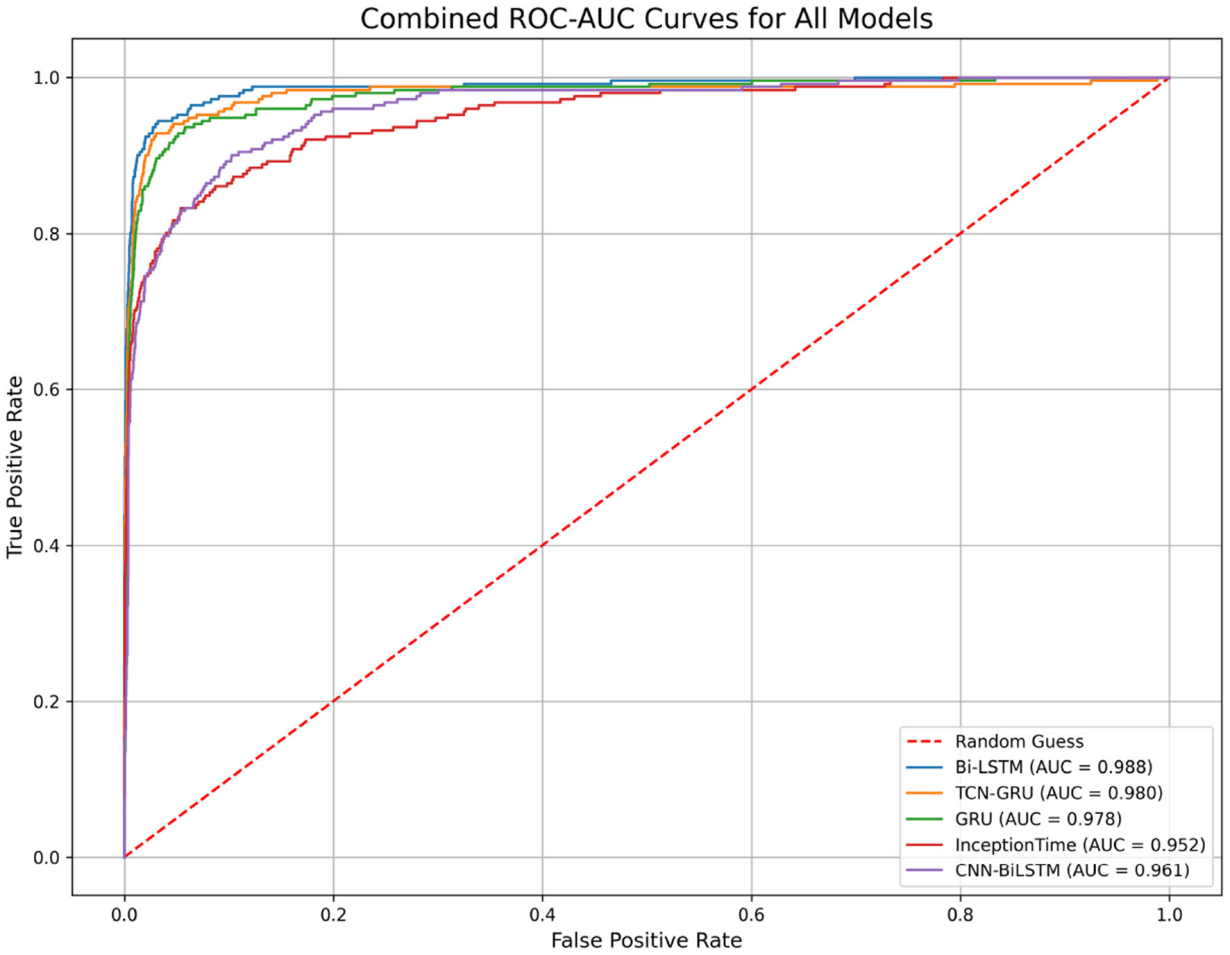
Combined Receiver Operating Characteristic (ROC) curves for all models. The Area Under the Curve (ROC-AUC) measures a model's ability to distinguish between classes. While all models perform well, the Bi-LSTM achieves the highest score, indicating superior discriminative power.

The trade-off between precision and recall for all models is visualized in the Precision-Recall (PR) curves shown in Figure 9. This plot confirms the superiority of the Bi-LSTM model, as its curve dominates the others by maintaining high precision over a wider range of recall values. The performance of all models is well above the “No-Skill” baseline, which is determined by the class prevalence, indicating that all architectures learned meaningful predictive patterns. The PR curve is particularly informative for imbalanced datasets, as it evaluates a model's ability to identify minority class instances without being skewed by the large number of true negatives(no pit stop). Figure 10 displays the Receiver Operating Characteristic (ROC) curves. While all models achieve high ROC-AUC scores, which can be optimistically skewed by the large true negative rate in imbalanced datasets, these results nonetheless confirm strong overall classification capability. The Bi-LSTM model again leads with the highest ROC-AUC score of 0.988. Table 5 shows the confusion matrix of the Bi-LSTM model on the test set with a threshold of 0.5277, reflecting the classification results across pit stop predictions.

**Table 5:** Bi-LSTM confusion matrix on test set (threshold = 0.5277).

**Actual\predicted**	**No Pitstop (0)**	**Has Pitstop (1)**
No Pitstop (0)	8,171	66
Has Pitstop (1)	35	216

**Figure 11:**
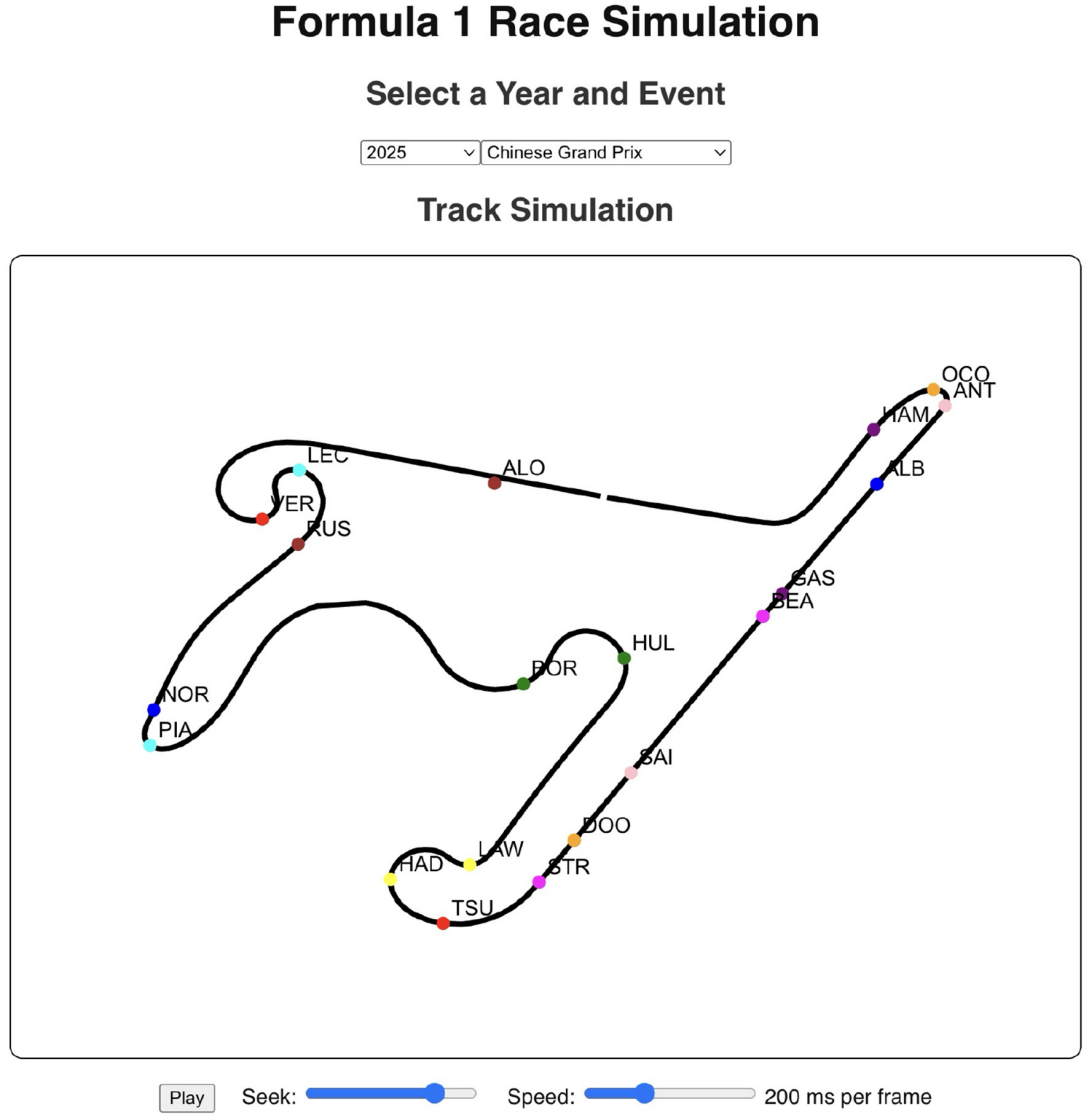
Race visualization interface for the 2025 Chinese Grand Prix. The visualization tool allows users to control playback speed, seek through frames, and view animated driver positions from the historical race data.

**Figure 12:**
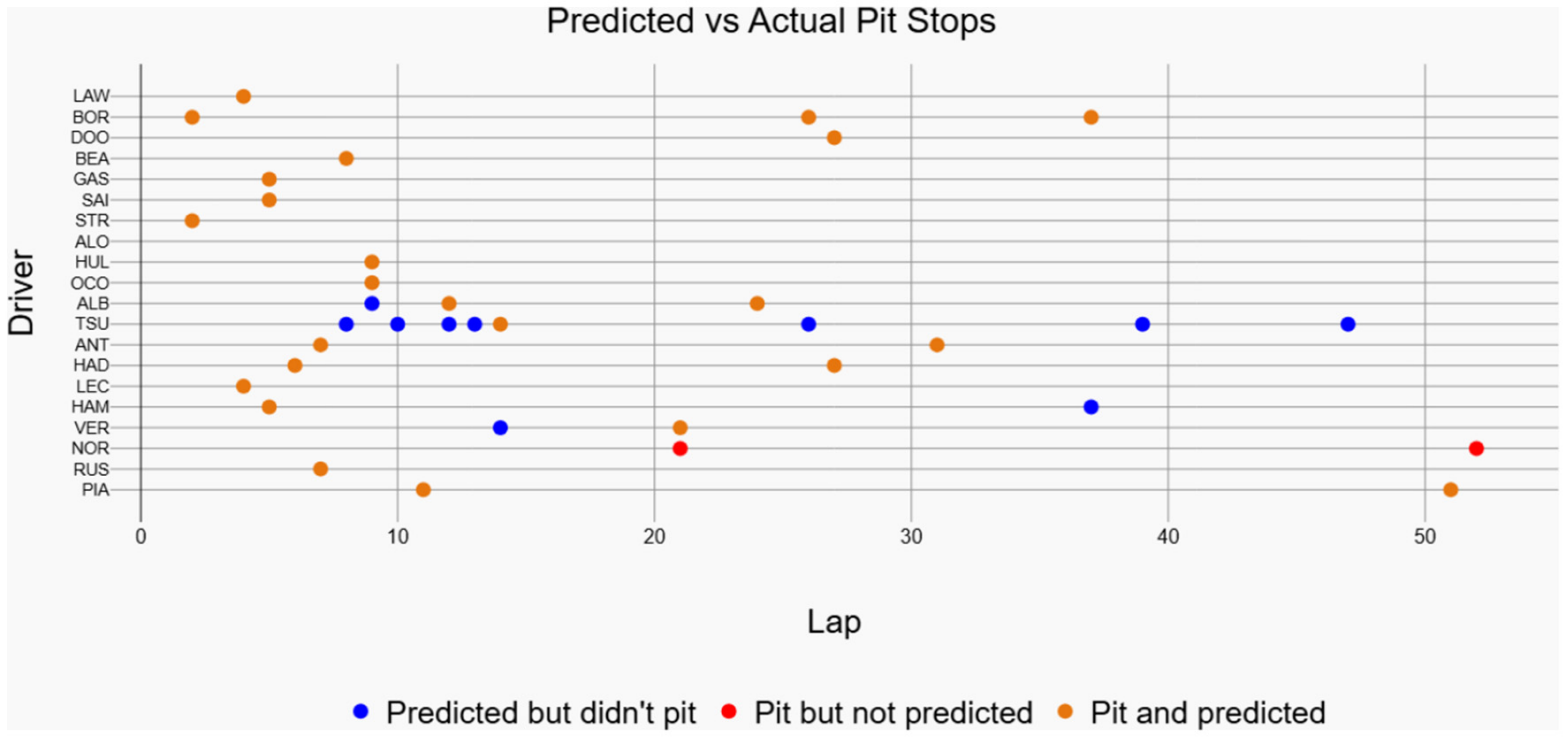
Predicted vs actual pit stops for each driver in the 2025 Chinese Grand Prix. Orange colored markers represent correctly predicted pit stops, blue markers denote predictions without actual pits, and red markers indicate actual pit stops missed by the model.

The historical race visualization interface is an analytical tool designed to replay and examine past Formula 1 race events using actual, real-world telemetry data. Unlike simulation tools that generate hypothetical scenarios, this interface provides a faithful reconstruction of historical races, animating the positions and movements of all drivers on the track throughout the event, which are available in FastF1 API. To demonstrate the model's practical performance, this interface was used to generate visualizations for the 2025 Chinese Grand Prix and the 2024 Singapore Grand Prix. The performance shown in Figure 11 corresponds to the 2025 Chinese Grand Prix data, where the model achieved an accuracy of 70.59%, a recall of 92.31%, and an F1-score of 80%. Figure 12 showcases driver-wise comparison of predicted and actual pit stops across race laps, highlighting prediction accuracy using color coded dots. The confusion matrix for the Bi-LSTM model on the Chinese Grand Prix is shown in Table 6, which indicates strong prediction accuracy on pit stop instances.

**Table 6:** Confusion matrix for the 2025 Chinese Grand Prix.

**Actual\predicted**	**Pred 0**	**Pred 1**
Actual 0	1,019	10
Actual 1	2	24

**Figure 13:**
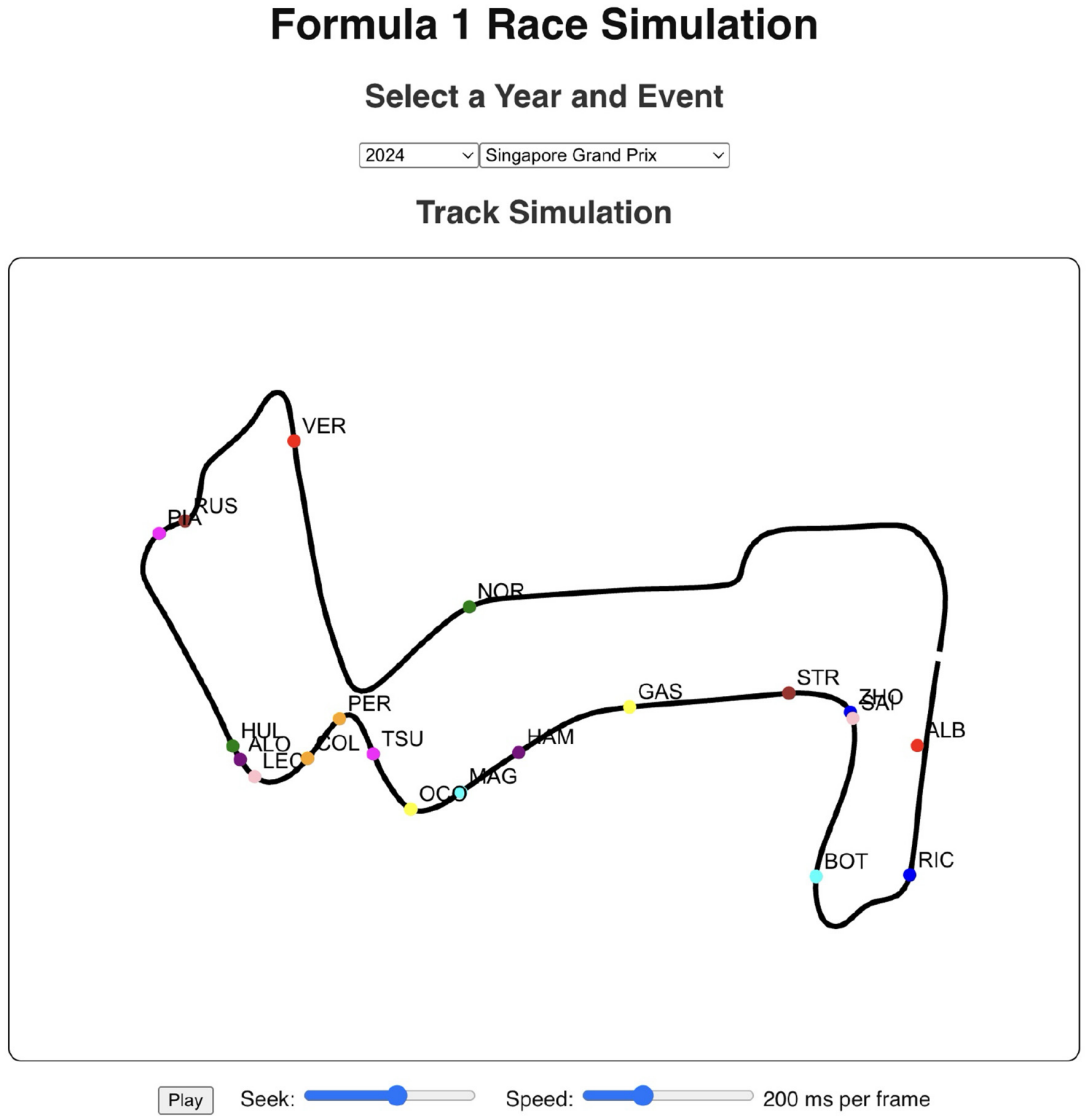
Race visualization interface for the 2024 Singapore Grand Prix. The interface visualizes driver positions on the Marina Bay Street Circuit using FastF1 telemetry data, with options to control playback speed and seek through race frames.

**Figure 14:**
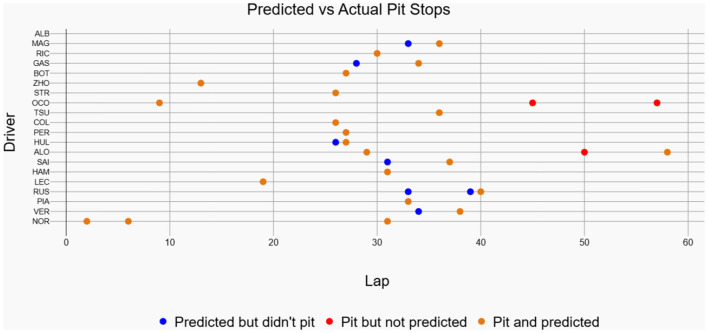
Predicted vs actual pit stops for each driver in the 2024 Singapore Grand Prix. Orange colored markers represent correctly predicted pit stops, blue markers denote predictions without actual pits, and red markers indicate actual pit stops missed by the model.

The 2024 Singapore Grand Prix results depicted in Figure 13 effectively demonstrate the historical race visualization interface's ability to visualize drivers navigating around the challenging Marina Bay Street Circuit. As shown in Figure 14, a color-coded comparison of predicted pit stops against actual stops provides insight into each driver's performance, distinguishing accurate forecasts from false positives and negatives. As summarized in Table 7, the model's confusion matrix for the race confirms its strong predictive reliability, correctly identifying 22 pit stop laps as well as 1135 non pit stop laps.

**Table 7:** Confusion matrix for the 2024 Singapore Grand Prix.

**Actual\predicted**	**Pred 0**	**Pred 1**
Actual 0	1,135	7
Actual 1	3	22

## 6 Conclusion

This study presents a deep-learning framework that predicts Formula 1 pit-stop timings directly from telemetry. After rigorous data-pre-processing and a comparative evaluation of several network architectures, the bidirectional LSTM proved most effective at capturing race dynamics. Integrated with an interactive historical race visualization interface, the model supplies real-time insights to support strategic decision-making.

Current constraints include limited access to high-fidelity telemetry and frequent regulatory changes during the season. Even so, the framework demonstrates the practical value of AI-driven decision support and offers a solid foundation for future motorsport applications.

## 7 Limitations and future scope

While this study demonstrates the potential of deep learning models to predict Formula 1 pit stop timings from publicly available telemetry data, several limitations must be acknowledged. These limitations primarily concern the impact of evolving regulations and the generalizability of models trained on public vs. proprietary team data. Addressing these challenges will be crucial for enhancing the external validity and practical applicability of future research.

### 7.1 Impact of evolving regulations

A significant challenge for any predictive model in Formula 1 is the sport's constantly evolving regulatory landscape. The Fédération Internationale de l'Automobile (FIA) frequently introduces changes to sporting and technical regulations that can substantially alter pit stop strategies. For instance, recent seasons have seen modifications to tire allocation, the introduction of sprint races with different pit stop rules, and even circuit-specific mandates, such as the new rule for the 2025 Monaco Grand Prix requiring two mandatory pit stops to increase strategic variability.

These regulatory shifts can render historical data less representative of current and future race conditions. A model trained on data from a period with a single mandatory pit stop, for example, may not generalize well to a season where multiple stops are required or where new tire compounds with different degradation characteristics are introduced. The dynamic nature of these rules means that predictive models must be continuously retrained and validated against the most recent data to maintain their accuracy and relevance.

### 7.2 Generalizability of models trained on publicly available data

Another key limitation of this study is its reliance on publicly available telemetry data, such as that provided by the FastF1 API. While this data is extensive, it represents only a fraction of the high-fidelity, real-time information available to the teams themselves. Formula 1 teams have access to proprietary data streams from hundreds of sensors on their cars, providing granular insights into tire temperature, brake wear, fuel load, and real-time car performance. This proprietary data allows teams to build much more sophisticated and accurate predictive models for pit stop optimization. Models trained on public data, therefore, may not capture the full complexity of the decision-making process on the pit wall. For example, a sudden increase in tire temperature or unexpected degradation that would trigger a pit stop for a team might not be visible in the public data stream. This discrepancy can lead to a model that is well-calibrated to the patterns in the public data but fails to predict pit stops that are triggered by factors only visible in the proprietary data.

### 7.3 Additional avenues for future research

The current model performs a binary classification (pit or no pit). A natural extension is a multi-class model that not only predicts if a pit stop is optimal but also recommends the ideal tire compound to switch to (e.g., Hard, Medium, or Soft), turning it into a more comprehensive decision support tool. A Reinforcement Learning framework could learn optimal pit stop policies by optimizing for long-term rewards (e.g., final race position or championship points) through simulation, potentially discovering counter-intuitive but highly effective strategies that are not apparent in historical data. Furthermore, a particularly promising direction for future research is the development of hybrid models. Such models could integrate the explicit strategic reasoning of game theoretic frameworks to model opponent interactions with the robust pattern recognition capabilities of deep learning models to process high dimensional telemetry data. This could lead to a decision support system that is both strategically aware and empirically grounded. Also, the proposed system is not restricted to just Formula 1 as there are multiple other motorsports where pit stops play an essential role in race strategy. These include but are not limited to MotoGP, NASCAR, IndyCar and Endurance racing events such as the twenty-four hours of LeMans.

## Data Availability

The original contributions presented in the study are included in the article/supplementary material, further inquiries can be directed to the corresponding author.
